# FAT10 is a Prognostic Biomarker and Correlated With Immune Infiltrates in Skin Cutaneous Melanoma

**DOI:** 10.3389/fmolb.2022.805887

**Published:** 2022-03-01

**Authors:** Yu Wang, Haiyue Zhang

**Affiliations:** ^1^ School of Laboratory Medicine and Life Science, Wenzhou Medical University, Wenzhou, China; ^2^ Department of Clinical Laboratory, The First Hospital of Jiaxing and The Affiliated Hospital of Jiaxing University, Jiaxing, China

**Keywords:** FAT10, bioinformatics analysis, tumor-infiltrating, prognosis, skin cutaneous melanoma, competing endogenous RNA

## Abstract

**Background:** Skin Cutaneous Melanoma (SKCM) is the deadliest cutaneous neoplasm. Previous studies have proposed ubiquitin-like protein FAT10 plays key roles in the initiation and progression of several types of human cancer, but little is known about the interrelation between *FAT10* gene expression, tumor immunity, and prognosis of patients with SKCM.

**Methods:** Here, we first performed pan-cancer analysis for FAT10’s expression and prognosis using the Cancer Genome Atlas and the Genotype-Tissue Expression data. Subsequently, we investigated the mRNA expression level, prognostic value, and gene-gene interaction network of FAT10 in SKCM using the Oncomine databases, GEPIA, TIMER, UALCAN, and starBase. The relationship between FAT10 expression and tumor immune invasion was studied by using the TIMER database. Additionally, the expression and functional status of FAT10 in SKCM were evaluated by the single-cell RNA sequencing and CancerSEA databases.

**Results:** In this study, we found that FAT10 expression was increased in SKCM and was correlated with a better survival rate in patients with SKCM. Moreover, we identified FAT10 level was significantly positively associated with immune infiltrates, biomarkers of immune cells, and immune checkpoint expression, and negatively correlated with tumor cell invasion and DNA damage, indicating that increased FAT10 expression in SKCM was a favorable response to immune checkpoint inhibitors.

**Conclusion:** Our findings suggest that upregulation of FAT10 correlated with better prognosis and tumor immune infiltration in SKCM.

## Introduction

Skin Cutaneous Melanoma (SKCM) is a primary malignant tumor that originates from benign moles (Cramer, 1991). It is derived from neural crest stem cells, which can produce melanin in the skin (Ding et al., 2021). Nevis will not develop into melanoma in most cases (Damsky and Bosenberg, 2017). However, certain factors increase the possibility of malignant transformation, such as Sun exposure, fair skin type, constant friction, or physical irritation to the affected skin (Bloethner et al., 2009; Darmawan et al., 2019). SKCM accounts for about 5% of the total number of skin cancers, but its death toll accounts for more than 75% of skin cancer deaths (Rebecca et al., 2020). The 5 years relative survival rate of local SKCM patients was 98%, the 5 years relative survival rate of regional SKCM patients was 64%, while the 5 years survival rate of patients with metastatic SKCM dropped to 23% (Rebecca et al., 2020). Therefore, we urgently need to clarify the molecular mechanism of SKCM, which may lead to the development of new treatments to improve long-term survival.

Fan *et al.* first discovered the ubiquitin-like protein FAT10 in 1996 (Fan et al., 1996). FAT10 shares many similarities with ubiquitin, while ubiquitin recycled from the degraded target proteins, FAT10 degraded along with its target, resulting in a relatively short half-life (Hipp et al., 2005; Liu et al., 2018). It is a protein belonging to the immune system, which can be strongly upregulated by pro-inflammatory cytokines (Aichem and Groettrup, 2016). Some reports described that FAT10 is involved in cardioprotection and regulates IRE1ɑ/c-Jun N-terminal kinase protein-dependent apoptosis in pancreatic β cells (Brozzi et al., 2016; Zhang et al., 2020). Animal studies have found that FAT10 “knockout” mice showed no distinct phenotypic changes, even if they do, they are relatively small. But these mice became more sensitive to endotoxin attack, and compared with wild-type mice, their lymphocytes were more prone to spontaneous apoptosis (Canaan et al., 2006). The description of the role of FAT10 in cancer in the published literature is contradictory. Several clinical studies have shown that FAT10 could confer malignant characteristics to non-tumorigenic cells and enhance the malignant-related characteristics of cancer cells (Gao et al., 2014; Zhang et al., 2020). However, the colony formation and transformation ability of FAT10 was not observed in the experiment of S Lukasiak *et al.*, which contradicts the function of FAT10 as a proto-oncogene (Lukasiak et al., 2008).

Despite these previous studies, there is still a lack of systematic research on the expression, prognosis, and mechanism of FAT10 in SKCM. In addition, the association between FAT10 and tumor immune infiltration in SKCM has not yet been determined. This study conducted expression analysis and survival analysis of FAT10 in various types of human cancers. Furthermore, the noncoding RNA (ncRNA)-associated regulation of FAT10 was explored in SKCM, including microRNA (miRNA) and long noncoding RNA (lncRNA). We then determined the relationship between FAT10 expression and immune cell infiltration, biomarkers of immune cells, or immune checkpoints in SKCM. Together, our findings corroborate that FAT10 may play a key role in the prognosis of SKCM while indicating a potential mechanism by which the FAT10 expression might adjust tumor immunity by regulating the infiltration of immune cells in SKCM patients.

## Materials and Methods

### Oncomine Database Analysis

Oncomine (https://www.oncomine.org/resource/login.html) is a comprehensive data mining platform, as well as the world’s largest cancer-related gene microarray database. It contains the most complete cancer mutation profile, related gene expression profile, and related clinical information, which can be used to discover new biomarkers or therapeutic targets. In this study, the *FAT10* gene was selected as the research object to compare its expression level in cancer tissues and normal tissues. When the folding change>1.5, with a *p*-value>0.001, the expression levels in different tissues were considered to be significantly different. We set the data type to “all” and the threshold value of gene rank to “top 10%” (Rhodes et al., 2007).

### Gene Expression Profiling Interactive Analysis Database Analysis

Gene Expression Profiling Interactive Analysis (GEPIA, www.gepia.cancer-pku.cn) is an interactive web server for cancer and normal gene-expression profiling and interactive analyses based on the Cancer Genome Atlas (TCGA) and Genotype-Tissue Expression (GTEx) data, with 9,736 tumors and 8,587 normal samples, respectively (Tang et al., 2017). GEPIA is used to analyze the expression of FAT10 and lncRNA in various types of cancer based on a given TCGA and GTEx expression data set, under the settings of the *p*-value of 0.01 and fold change of 1. The impact of FAT10 expression on survival rates was evaluated using the GEPIA, including overall survival (OS) and disease-free survival (RFS). GEPIA was also employed to conduct the prognostic values of candidate lncRNAs in FAT10. A log-rank *p*-value <0.05 was considered statistically significant. Moreover, FAT10 expression correlation with immune checkpoints in SKCM was perform using the GEPIA database. Items with ΙRΙ >0.1 and *p*-value <0.05 were set as selection criteria and determined to be significant.

### Candidate miRNA Prediction

The upstream binding miRNA of FAT10 was predicted by multiplel target gene prediction programs (PITA, RNA22, miRmap, microT, miRanda, PicTar, and TargetScan). When predictive miRNAs appeared in more than two programs at the same time, they were used for subsequent analysis. These predicted miRNAs were considered candidate miRNAs of FAT10.

### UALCAN Database Analysis

The UALCAN portal (http://ualcan.path.uab.edu/analysis-prot.html) is an interactive network resource that can be used to analyze cancer omics data (Chen et al., 2019). We used this online tool to assess the expression level of the upstream binding miRNAs of FAT10 of SKCM located in metastases, primary tumors, and normal tissues.

### starBase Database Analysis

starBase (http://starbase.sysu.edu.cn/), a database for exploring miRNA-related research (Li et al., 2014). In our study, starBase was introduced to perform survival analysis on hsa-miR-3127-5p in SKCM. A log-rank *p*-value <0.05 was considered statistically significant. In addition, we predicted candidate lncRNAs that might bind to hsa-miR-3127-5 based on starBase.

### TIMER Database Analysis

TIMER (https://cistrome.shinyapps.io/timer/) is a web server for determining the abundance of tumor infiltrates based on gene expression analysis (Li et al., 2017). The gene name *FAT10* under the DiffExp module has default parameters was used for obtaining the different expression levels in normal or tumor tissues. We used TIMER to analyze the level of immune cell infiltration in SKCM and the correlation between FAT10 and tumor-infiltrating immune cell biomarker gene expression, including B cells, CD8^+^ T cells, CD4^+^ T cells, M1 macrophages, M2 macrophages, neutrophils, and dendritic cells. *p*-value <0.05 was considered statistically significant.

### Human Protein Atlas and CancerSEA Database Analysis

To confirm our outcome, The Human Protein Atlas (www.proteinatlas.org/) was utilized to determine the expression of FAT10 at the translational level (Thul et al., 2017). The expression and distribution of FAT10 in SKCM tissues was clarified by the single-cell RNA sequencing (scRNA-seq), which was obtained from CancerSEA (Yuan et al., 2019).

### Statistical Analysis

All data analyses was automatically calculated by the above-mentioned online database. Log-rank *p*-value <0.05 or *p*-value <0.05 were considered statistically significant.

## Results

### Expression of FAT10 in SKCM and Other Cancers

**FIGURE 1:**
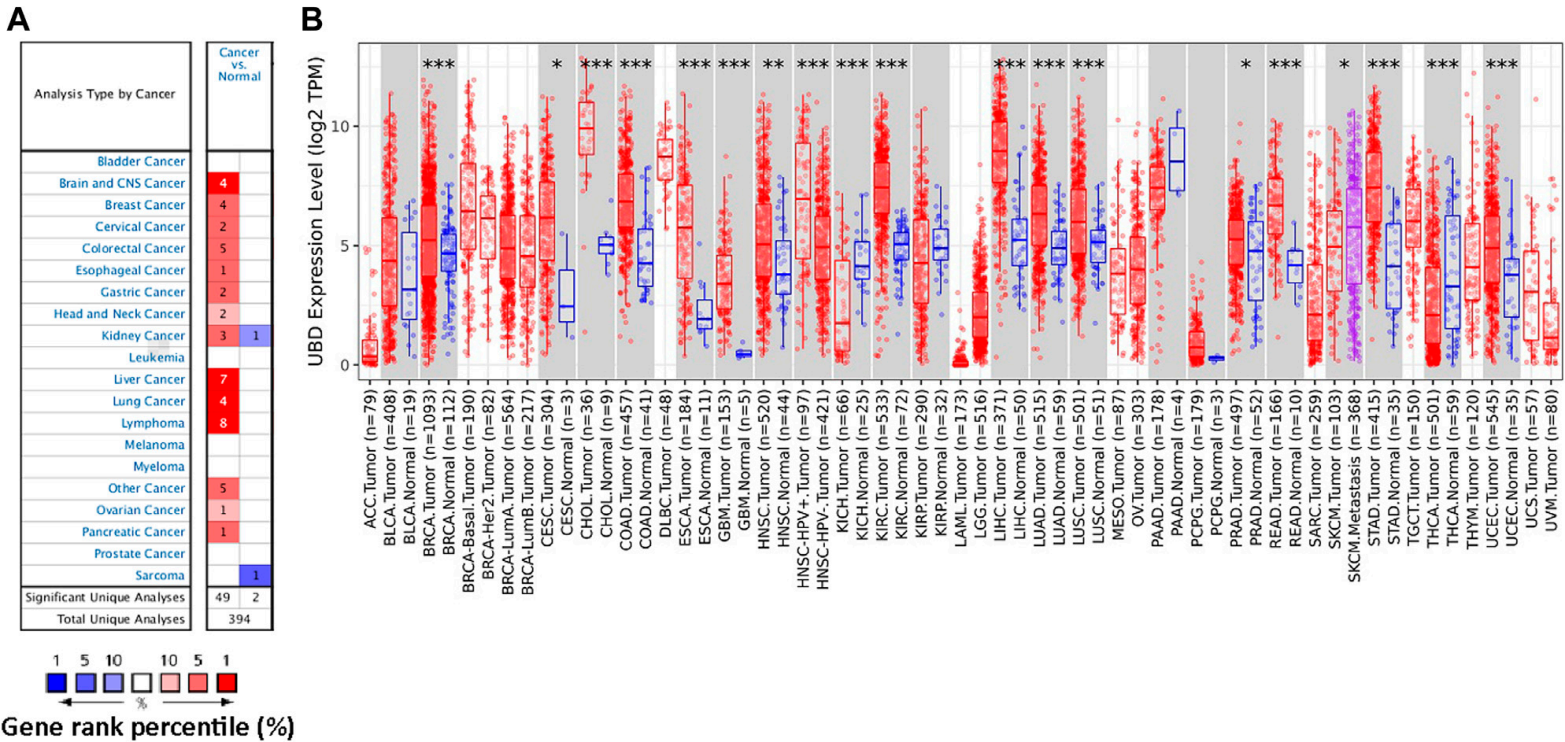
The expression level of *FAT10* in various cancer tissues or tumor cells. **(A)** Through Oncomine analysis, the expression level of *FAT10* in various normal or cancer tissues. **(B)** Through TIMER analysis, the expression level of *FAT10* in various normal or cancer tissues (*p < 0.05, ***p < 0.001).

In order to study the possible role of FAT10 in cancer, we first analyzed its expression in various cancer types through Oncomine database analysis. Except for sarcoma cancer, compared with normal tissues, the expression level of *FAT10* gene in all other available cancer tissues was significantly up-regulated, including brain and central nervous system cancer, breast, cervical, colorectal, esophageal, gastric, head and neck, liver, lung, lymphoma, ovarian, kidney, and pancreatic tissues (Figure 1A). Similarly, the RNA-seq data we collected from the TCGA database showed that significantly up-regulated *FAT10* was detected in 19 types of cancers, including SKCM (Figure 1B). We also collected RNA-seq data from the TCGA and GTEx expression data sets, and the results showed that *FAT10* was significantly up-regulated in 21 cancer types (Supplementary Figure S1).

### The Prognostic Values of FAT10 in Human Cancer

**FIGURE 2:**
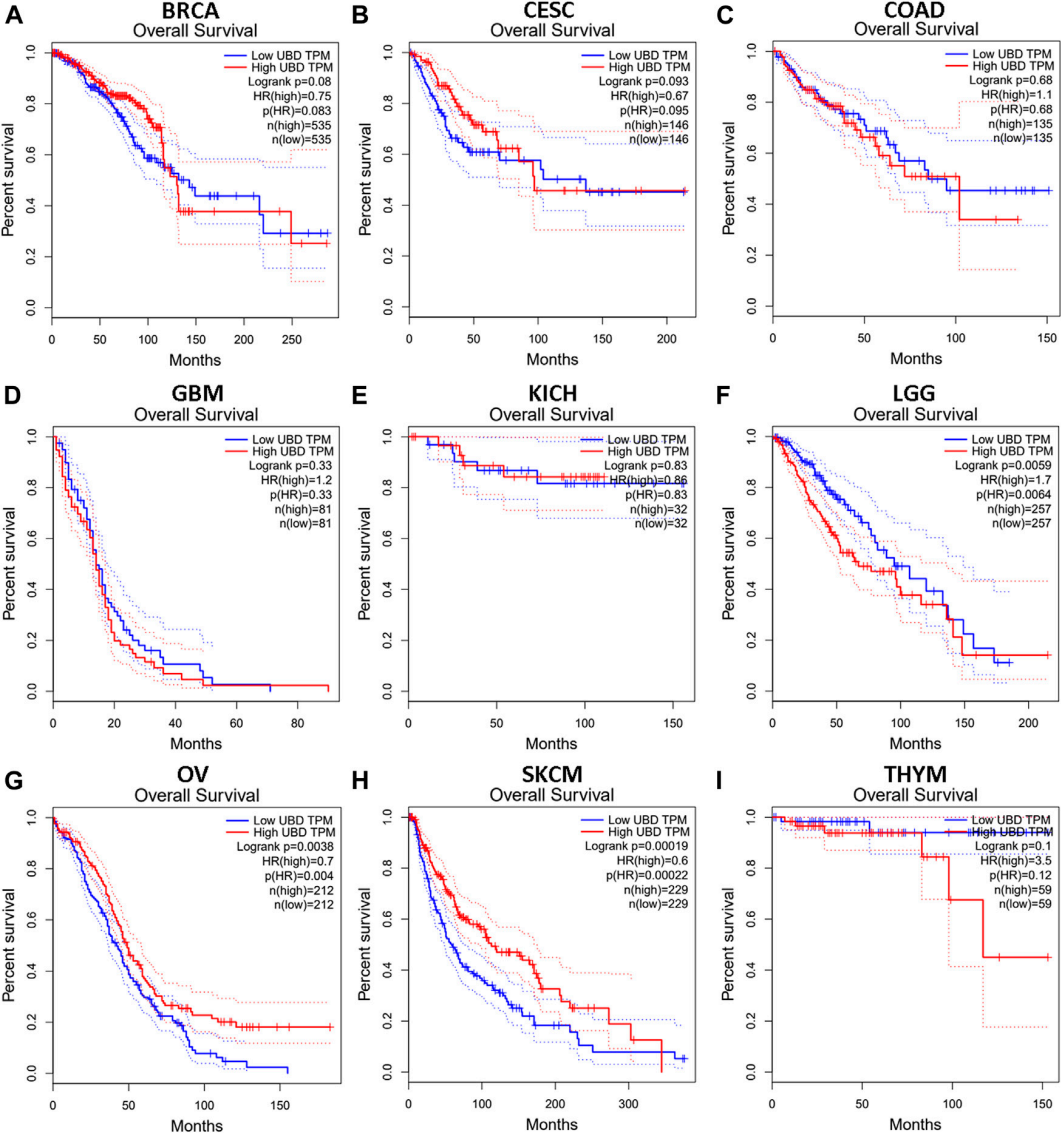
The OS analysis for FAT10 in various human cancers via GEPIA database. **(A–I)** The OS plot of FAT10 in BRCA **(A)**, CESC **(B)**, COAD **(C)**, GBM **(D)**, KICH **(E)**, LGG **(F)**, OV **(G)**, SKCM **(H)**, THYM **(I)**.

**FIGURE 3:**
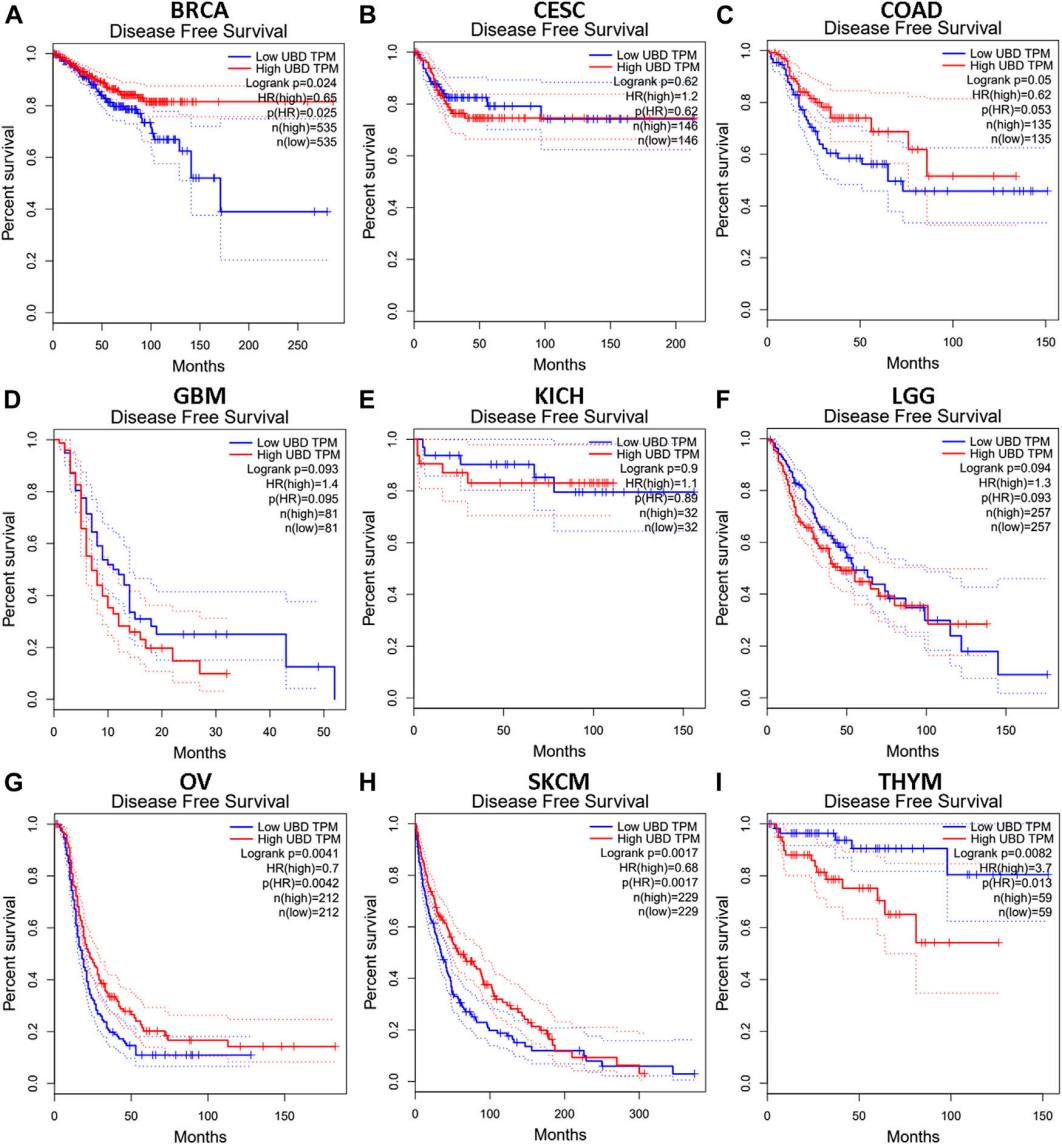
The RFS analysis for FAT10 in various human cancers via GEPIA database. **(A–I)** The RFS plot of FAT10 in BRCA **(A)**, CESC **(B)**, COAD **(C)**, GBM **(D)**, KICH **(E)**, LGG **(F)**, OV **(G)**, SKCM **(H)**, THYM **(I)**.

We investigated whether the expression level of FAT10 affected the prognosis of cancer patients (BRCA, CESC, COAD, GBM, KICH, LGG, OV, SKCM, and THYM). Two prognostic indices, consisting of OS and RFS, were included. For OS, highly expressed FAT10 in OV and SKCM patients had a better prognosis, but LGG patients with higher expression of FAT10 indicated an unfavorable prognosis (Figure 2). For RFS, high FAT10 expression levels were associated with better prognosis in BRCA, OV, and SKCM patients, but THYM patients with higher expression of the *FAT10* gene were related to poor prognosis (Figure 3). We have not observed that FAT10 was statistically significant in predicting the prognosis of patients in other types of cancer. These results confirmed the prognostic value of FAT10 in certain types of cancer, and the increase or decrease of FAT10 expression has different prognostic values, depending on the type of cancer.

### Prediction and Analysis of Upstream miRNAs of FAT10

ncRNA is responsible for the regulation of gene expression, which has been widely recognized. In order to determine whether FAT10 was regulated by some ncRNAs, we first predicted the upstream miRNAs that might bind to FAT10 and found 7 miRNAs.

**FIGURE 4:**
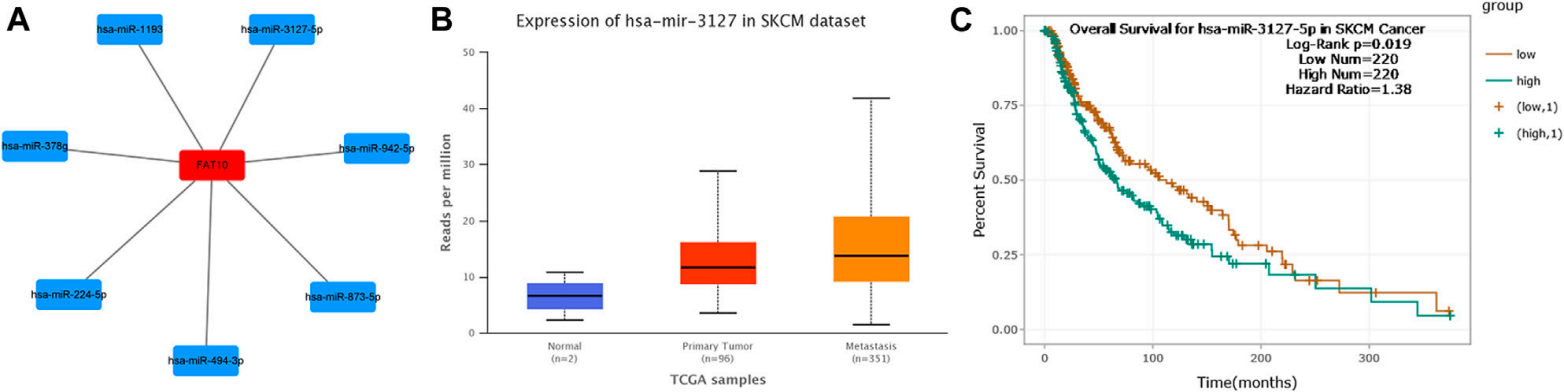
Identify hsa-miR-3127-5p as a potential upstream miRNA of FAT10 in SKCM. **(A)** The miRNA-FAT10 regulatory network was established via cytoscape software. **(B)** The expression of hsa-miR-3127-5p in SKCM and control normal samples via UALCAN analysis. **(C)** The prognostic value of hsa-miR-3127-5p in SKCM assessed by starBase database.

To visualize the results, we used cytoscape software to establish a miRNA-FAT10 regulatory network. According to the mechanism of miRNA regulating target gene expression, there should be a positive correlation between miRNA and FAT10. Therefore, we conducted a related expressive analysis. As shown in Figure 4, there is a significant positive correlation between FAT10 and hsa-miR-3127-5p in SKCM. No statistical expression relationship between FAT10 and the other six predicted miRNAs was observed. We finally determined the expression and prognostic value of hsa-miR-3127-5p in SKCM.

### Prediction and Analysis of Upstream lncRNAs of Hsa-miR-3127-5p

**FIGURE 5:**
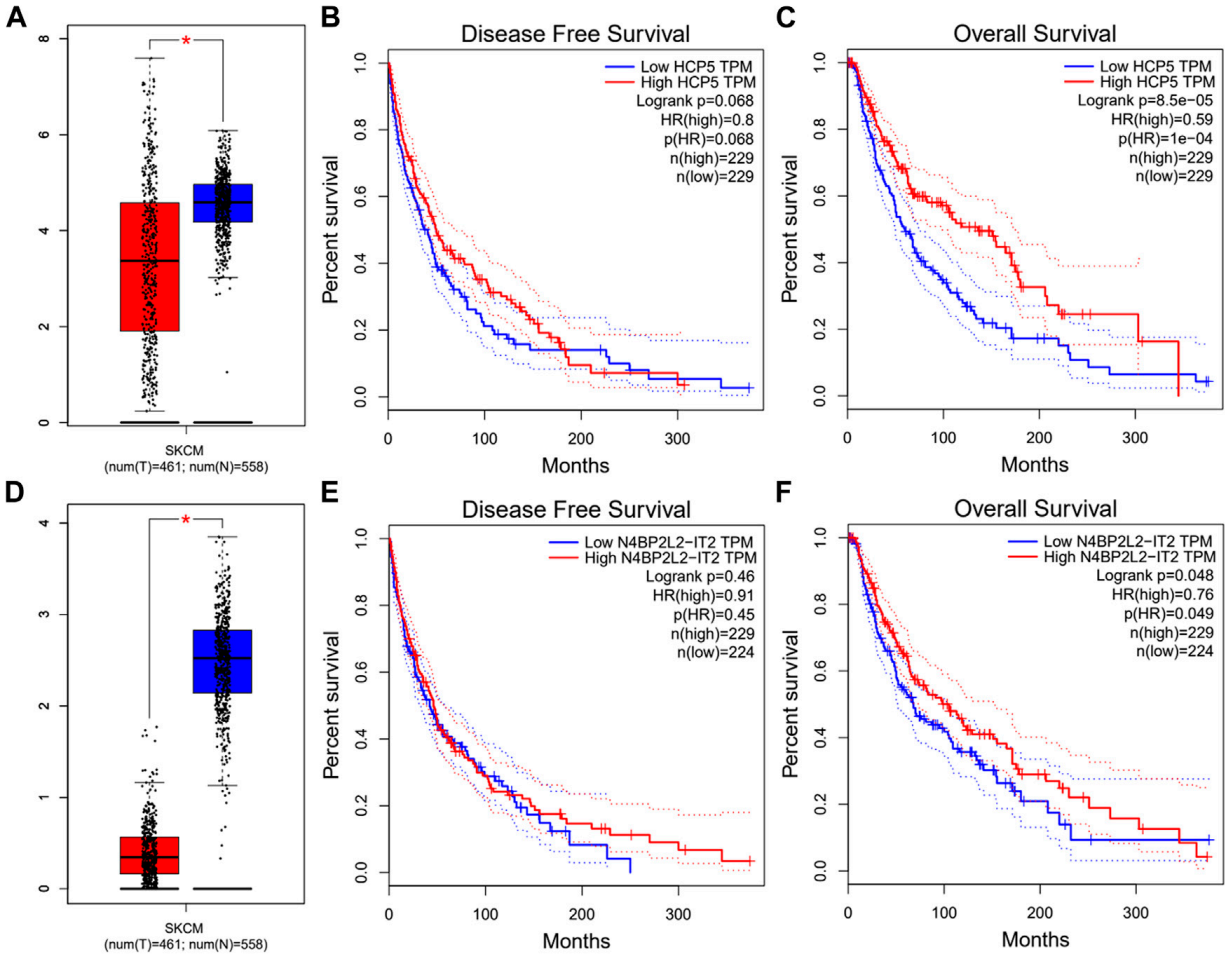
Expression analysis and survival analysis of lncRNA upstream of hsa-miR-3127-5p in SKCM. **(A,D)** The expression of HCP5 **(A)** and N4BP2L2-IT2 **(D)** in TCGA SKCM compared with “TCGA and GTEx normal” data. **(B,C,E,F)** The RFS analysis for HCP5 **(B)** and N4BP2L2-IT2 **(E)** in HCC. The OS for HCP5 **(C)** and N4BP2L2-IT2 **(F)** in SKCM. *p-value < 0.05.

Next, we used the starBase database to predict the upstream lncRNA of hsa-miR-3127-5p. There were 115 possible lncRNAs discovered. Futuremore, GEPIA was used to determine the expression level of these lncRNAs in SKCM. We found that among all 115 lncRNAs in SKCM, 20 lncRNAs were significantly down-regulated compared with the normal control, including HCP5 and N4BP2L2-IT2. Subsequently, we evaluated the prognostic value of 20 lncRNAs in SKCM. As suggested in Figure 5, SKCM patients with higher expression of HCP5 or N4BP2L2-IT2 possessed better OS. The competitive endogenous RNA (ceRNA) hypothesis suggests that lncRNA can increase mRNA expression through competitive binding with shared miRNAs. Thus, there should be a positive correlation between lncRNA and mRNA or a negative correlation between lncRNA and miRNA. We also used the starBase database to detect the expression correlation between lncRNA and hsa-miR-3127-5p/FAT10 in SKCM, as shown in Table 1. Considering the results of expression analysis, survival analysis, and correlation analysis, HCP5 might be the most potential upstream lncRNA of the hsa-miR-3127-5p/FAT10 axis in SKCM.

**TABLE 1:** Correlation analysis between lncRNA and hsa-miR-3127-5p or lncRNA and FAT10 in SKCM via starBase database.

lncRNA	miRNA	R value	*p* value
HCP5	hsa-miR-3127-5p	−0.220^a^	2.49E-06^***,^ ^a^
N4BP2L2-IT2	hsa-miR-3127-5p	0.100^a^	3.02E-02^*,^ ^a^
lncRNA	mRNA	R value	*p* value
HCP5	FAT10	0.690^a^	4.70E-67^***,^ ^a^
N4BP2L2-IT2	FAT10	0.120^a^	1.00E-02^*,^ ^a^

a These results are statistically significant.

*p value < 0.05; ***p value < 0.001.

### FAT10 Positively Correlates With Immune Cell Infiltration in SKCM

**FIGURE 6:**
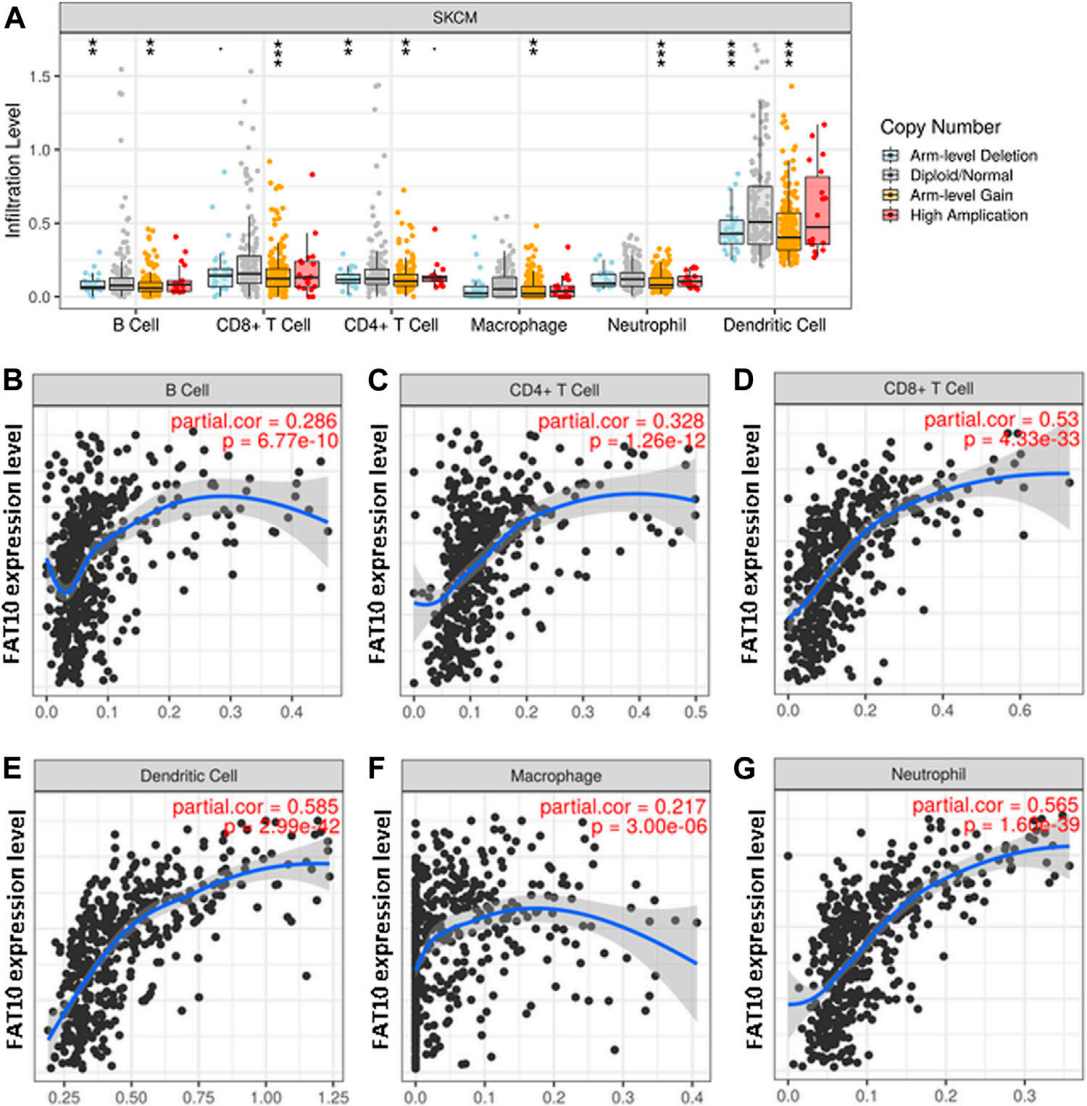
The relationship between immune cell infiltration and FAT10 level in SKCM. **(A)** The infiltration level of various immune cells in SKCM with different copy numbers of FAT10. **(B–G)** FAT10 expression level and B cells **(B)**,CD4^+^ T cells **(C)**, CD8^+^ T cells **(D)**, dendritic cells **(E)**, macrophages **(F)**, or neutrophils **(G)** correlation of infiltration level in SKCM.

FAT10 is a member of ubiquitin-like proteins, which is involved in the inflammatory response and immune cell infiltration. We found that higher expression levels of FAT10 were related to better prognosis and high immune infiltration in SKCM. There were significant changes in the level of immune cell infiltration under different copy numbers of FAT10 (Figure 6A). The study of the correlation between the expression level of FAT10 and the level of immune cell infiltration could provide useful clues for studying the function and mechanism of FAT10. We then assessed the correlation between the expression level of FAT10 and the level of immune cell infiltration. As shown in Figures 6B–G, FAT10 expression was significantly positive correlation with all analyzed immune cells in SKCM (B cell, CD8^+^T cell, CD4^+^T cell, macrophage, neutrophil, and dendritic cell). These findings strongly indicate that FAT10 play a specific role in the immune infiltration of SKCM.

### Expression Correlation of FAT10 and Biomarkers of Immune Cells in SKCM

To investigate the role of FAT10 in tumor immunity, we used the GEPIA database to determine the correlation between FAT10 and the expression of immune cell biomarkers in SKCM. We found that FAT10 expression was positively correlated with B cell’s biomarkers (CD19 and CD79A), CD8^+^T cell’s biomarkers (CD8A and CD8B), CD4^+^T cell’s biomarker (CD4), M1 macrophage’s biomarkers (IRF5), M2 macrophage’s biomarkers (CD163, VSIG4, and MS4A4A), neutrophil’s biomarkers (ITGAM and CCR7), and dendritic cell’s biomarkers (HLA-DPB1, HLA-DQB1, HLA-DRA, HLA-DPA1, CD1C, NRP1, and ITGAX) in SKCM (Table 2). These results further indicate that there is a positive relationship between FAT10 and immune cell infiltration.

**TABLE 2:** Correlation analysis between FAT10 and biomarkers of immune cells in SKCM via GEPIA database.

Immune cell	Biomarker	R Value	*p* value
B cell	CD19	0.61^a^	7.8E-48^***,^ ^a^
	CD79A	0.66^a^	1.4E-58^***,^ ^a^
CD8^+^ T cell	CD8A	0.83^a^	1.5E-119^***,^ ^a^
	CD8B	0.82^a^	4.5E-115^***,^ ^a^
CD4^+^ T cell	CD4	0.68^a^	9.2E-63^***,^ ^a^
M1 macrophage	NOS2	−0.033	4.8E-01^a^
	IRF5	0.46^a^	6.9E-26^***,^ ^a^
	PTGS2	0.013	7.8E-01
M2 macrophage	CD163	0.54^a^	4.4E-36^***,^ ^a^
	VSIG4	0.53^a^	1.8E-34^***,^ ^a^
	MS4A4A	0.57^a^	2.6E-40^***,^ ^a^
Neutrophil	CEACAM8	-0.07	1.3E-01
	ITGAM	0.57^a^	1.3E-41^***,^ ^a^
	CCR7	0.71^a^	5.9E-72^***,^ ^a^
Dendritic cell	HLA-DPB1	0.78^a^	1.7E-94^***,^ ^a^
	HLA-DQB1	0.60^a^	1.3E-46^***,^ ^a^
	HLA-DRA	0.81^a^	1.8E-107^***,^ ^a^
	HLA-DPA1	0.79^a^	4.3E-100^***,^ ^a^
	CD1C	0.51^a^	2E-32^***,^ ^a^
	NRP1	0.14^a^	2.9E-03^**,^ ^a^
	ITGAX	0.49^a^	3.8E-29^***,^ ^a^

a These results are statistically significant.

**p value < 0.01; ***p value < 0.001.

### Relationship Between FAT10 and Immune Checkpoints in SKCM

**FIGURE 7:**
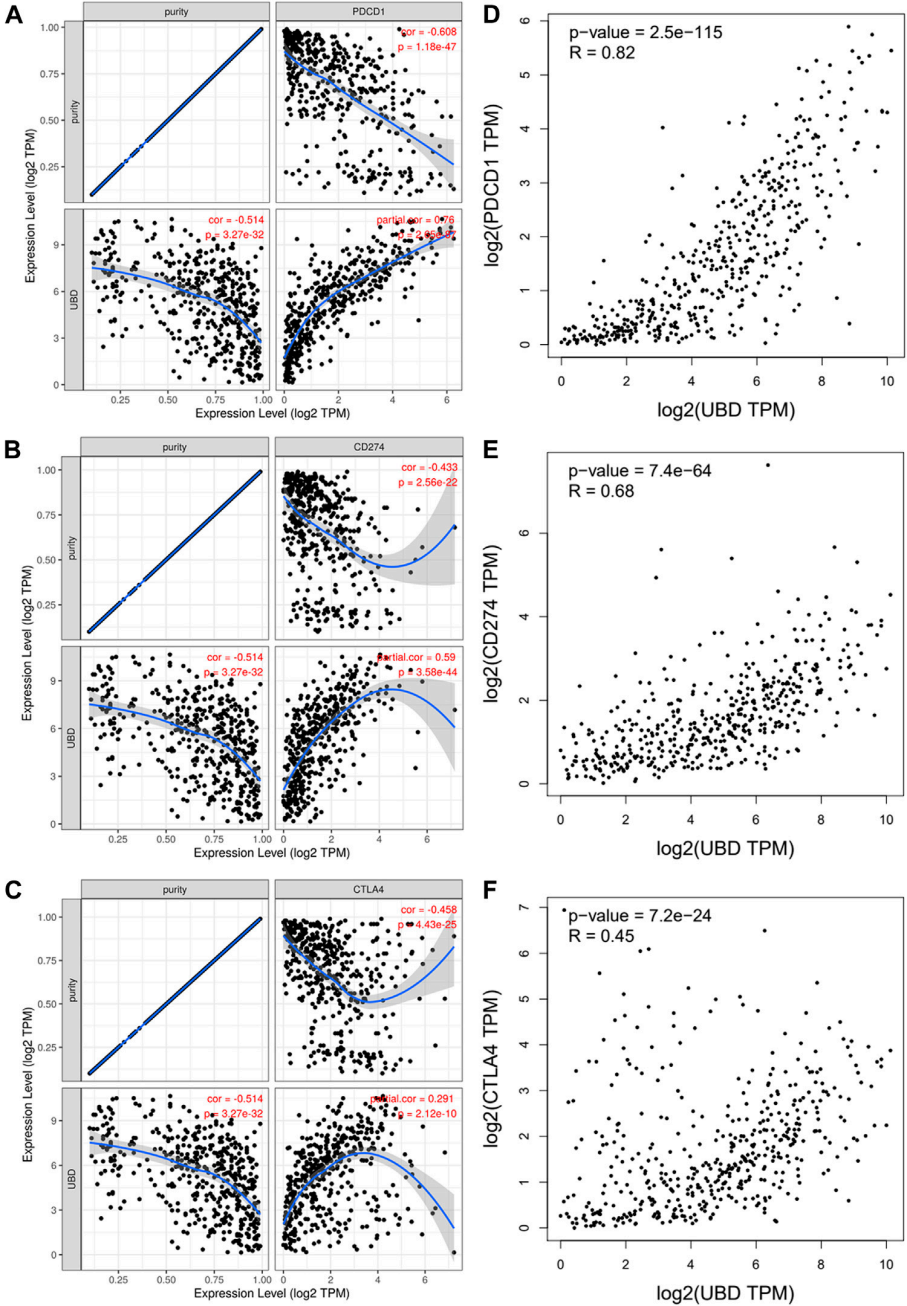
The correlation between FAT10 expression and PD-1, PD-L1 and CTLA-4 expression in SKCM. **(A)** Use TIMER to adjust the spearman correlation between FAT10 and PD-1 expression in SKCM through purity. **(B)** Use TIMER to adjust the spearman correlation between FAT10 and PDL1 expression in SKCM through purity. **(C)** Use TIMER to adjust the spearman correlation between FAT10 and CTLA-4 expression in SKCM through purity. **(D)** The expression correlation of FAT10 and PD1 in SKCM *via* GEPIA analysis. **(E)** The expression correlation of FAT10 and PD-L1 in SKCM via GEPIA analysis. **(F)** The expression correlation of FAT10 and CTLA-4 in SKCM *via* GEPIA analysis.

PD1 and CTLA-4 are important immune checkpoints, as well as negative regulators of T cell immune function. PD-L1 expression is present in many different tumor types, which is associated with a poor prognosis. Inhibition of these targets can increase activation of the immune system *in vivo*. Considering that FAT10 may play an important role in the development of SKCM, we evaluated the relationship between FAT10 and PD1, PD-L1, or CTLA-4. After purity adjustment, FAT10 in SKCM was significantly positively correlated with PD1, PD-L1, or CTLA-4 (Figures 7A–C). Similar to TIMER data analysis, GEPIA data analysis showed that FAT10 had a significant positive correlation with PD1, PD-L1, or CTLA-4 in SKCM (Figures 7D–F). These results indicate that the rational application of CTLA-4 and PD-1 or its ligand PD-L1 inhibitors can help restore the anti-tumor immune response, thereby bringing long-term benefits to patients.

### Validation of FAT10 Expression

**FIGURE 8:**
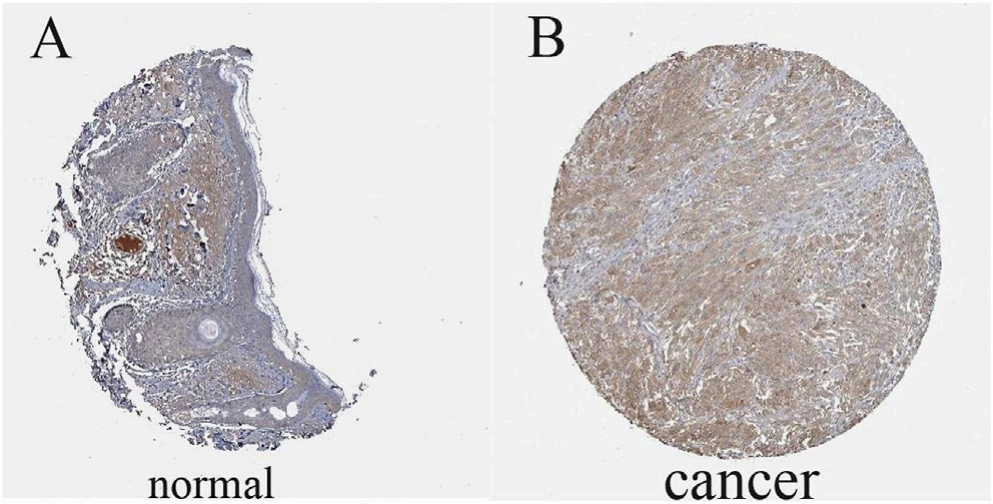
The level of FAT10 in normal skin tissues **(A)** and SKCM tissues **(B)**.

**FIGURE 9:**
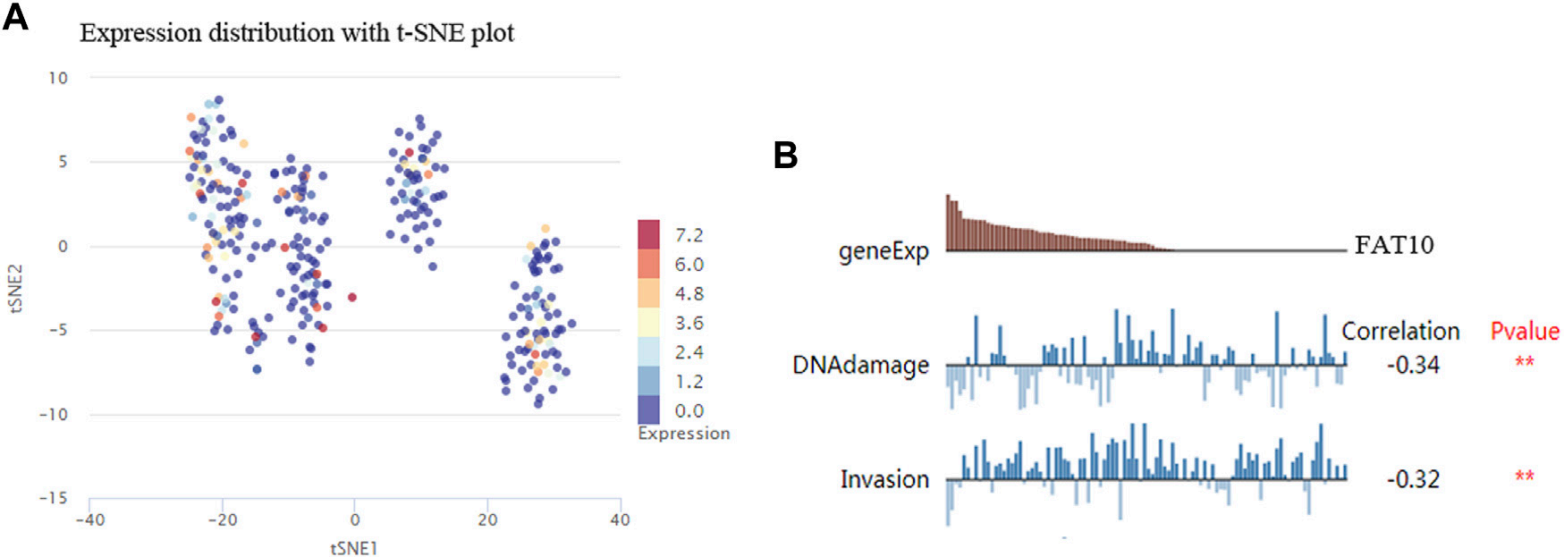
T-SNE describes the expression profile of FAT10 in a single cell obtained from SKCM tissue. Each dot represents a cell **(A)**. The CancerSEA database determines the correlation between FAT10 expression and two functional states **(B)**. **p < 0.01.

The results illustrated that the protein level of FAT10 was higher in SKCM tissues than in normal skin tissues (Figures 8A–B). T-SNE describes the expression profles of FAT10 in the single cells obtained from SKCM tissues. Each point represents a single cell (Figure 9A). We used CancerSEA to predict the FAT10-associated functional states in SKCM by scRNA-seq datasets, The result shows that FAT10 expression is negatively associatered with tumor cell invasion and DNA damage (Figure 9B).

## Discussion

Melanoma is a fatal skin cancer that affects many people around the world every year (Owens, 2014). In addition, due to the high resistance of metastatic melanoma to radiotherapy and chemotherapy drugs, its prognosis is extremely poor (Liu et al., 2012). Elucidating the molecular mechanism and process of SKCM may provide new ideas and insights for the development of effective therapeutic targets or the search for promising prognostic biomarkers. There is increasing evidence that has demonstrated that the ubiquitin-like modifier FAT10 is directly involved in the development of a variety of cancers, including SKCM. Nevertheless, the knowledge of FAT10 in SKCM is still inadequate, and further research is needed.

In this study, we first used the Oncomine database to perform pan-cancer analysis on the expression of FAT10, and then further verified the expression of FAT10 using the TIMER, GEPIA, and Human Protein Atlas database. The results of our FAT10 survival analysis for cancer types of interest show that FAT10 expression was increased in SKCM and correlated with a better survival rate in patients with SKCM. In addition, FAT10 expression is negatively associatered with tumor cell invasion and DNA damage. The research of Canaan *et al.* showed the pro-survival effect of FAT10 (Canaan et al., 2006). This report, together with our analysis, reveals the pro-survival effect of FAT10 in SKCM.

Although ncRNAs cannot encode proteins, they are ubiquitous in organisms (Yamamura et al., 2018). In the last 2 decades, a large number of studies have shown that ncRNA (including miRNA, lncRNA, and circular RNA) communicate with each other through the ceRNA mechanism and play a critical role in the regulation of gene expression (Gao et al., 2020; Ghafouri-Fard et al., 2020; Lou et al., 2020). To explore the upstream regulatory miRNAs of FAT10, seven prediction programs (PITA, RNA22, miRmap, microT, miRanda, PicTar, and TargetScan) were introduced to predict miRNAs that may bind to FAT10. In the end, we obtained seven miRNAs. The specific mechanisms of most of these miRNAs in SKCM were unknown or controversial. Previous studies have shown that hsa-miR-873-5p inhibits the translation of endogenous growth differentiation factor 15 (GDF15) in melanoma cell lines (Teng et al., 2017). However, the role of GDF-15 is still controversial, depending on the tumor entity and model studied (Baek and Eling, 2019). Based on the results of correlation analysis, expression analysis and survival analysis, hsa-miR-3127-5p was identified as the most potential upstream oncogenic miRNA of FAT10.

According to the ceRNA hypothesis theory (Salmena et al., 2011), the potential lncRNA of hsa-miR-3127-5p/FAT10 axis should be the tumor suppressor lncRNA in SKCM. Then, we predicted the upstream lncRNA of the hsa-miR-3127-5p/FAT10 axis. A total of 115 possible lncRNAs were discovered. Combining the results of expression analysis, survival analysis and correlation analysis, we finally determined the two most potential down-regulated lncRNAs (HCP5 and N4BP2L2-IT2). The two lncRNAs were involved in multiple immune-related processes, and cell proliferation, apoptosis (Zhou et al., 2021; Zou and Chen, 2021). The high level of HCP5 expression was related to the better survival of SKCM patients, could significantly inhibit the proliferation, colony formation, and invasion of primary SKCM cells and promote cell apoptosis. In contrast, low-level expression of HCP5 was associated with poor survival of SKCM patients (Zou and Chen, 2021). Finally, the HCP5 and N4BP2L2-IT2/hsa-miR-3127-5p/FAT10 axis were identified as potential regulatory pathways in SKCM.

A large number of studies have shown that tumor immune cell infiltration can affect the prognosis of cancer patients and the efficacy of radiotherapy, chemotherapy, or immunotherapy (Waniczek et al., 2017; Zhang et al., 2018; Lyu et al., 2020). To gain further insight into the mechanism of FAT10 in SKCM tumorigenesis, we performed the correlation between *FAT10* gene expression and immune cell infiltration. Our work suggested that the transcription levels of FAT10 was closely correlated with various levels of immune infiltration in SKCM. There is a moderate positive relationship between FAT10 expression level and infiltration level of macrophages, and significantly positive correlations between infiltration level of B cells, CD8^+^ T cells, CD4^+^ T cells, neutrophils, and dendritic cells. The *FAT10* gene structurally belongs to the major histocompatibility complex locus, which is composed of multiple genes that play different key roles in immune surveillance against cancer diseases effect (Gruen and Weissman, 1997; Canaan et al., 2006). At the tissue level, high-level expression of human FAT10 was found in lymphoid organs like the thymus, spleen, and lymph nodes(Lee et al., 2003; Lukasiak et al., 2008). FAT10 mRNA is expressed in organs where lymphocytes develop into mature and active, indicating that FAT10 protein may play a key role in lymphocyte maturation (Bates et al., 1997; Canaan et al., 2006). At the same time, in the process of pro-inflammatory immune response in the tumor microenvironment, the expression of FAT10 increases under the action of factors such as the release of pro-inflammatory factors by infiltrating macrophages (Lukasiak et al., 2008). FAT10 may in turn promotes the maturation of more lymphocytes. This may be the reason why the expression level of FAT10 is positively correlated with the infiltration of immune cells. Another important aspect of this study was the significant positive correlation between FAT10 and these biomarkers of infiltrating immune cells. Together, our findings suggested FAT10 might be involved in the regulation of SKCM tumor immunity.

In recent years, people have increasingly realized the role of the immune system in the occurrence and development of cancer, and at the same time, tumor immunotherapy has developed rapidly (Xie et al., 2021). Both sufficient immune cells infiltrate the tumor microenvironment and adequate expression of immune checkpoints were required for tumor immunotherapy to achieve curative effect (Chae et al., 2018). The CTLA-4 and PD-1 immune checkpoint pathways can maintain peripheral tolerance by down-regulating T cell activation, which can be used by tumors to grow and develop rather than being eliminated by the immune system. The use of CTLA-4 and PD-1 or its ligand PD-L1 inhibitor can relieve the immunosuppressive state and restore the anti-tumor immune response. Moreover, combined use of an anti-CTLA-4 immune-checkpoint inhibitor with an anti-PD-1/PD-L1 monoclonal antibody may have a complementary effect(Curran et al., 2010), leading to long-term benefit in a substantial proportion of patients receiving treatment (Buchbinder and Desai, 2016). In addition, when tumor-infiltrating immune cells express PD-L1, patients have the strongest response to anti-PD-L1 blockers (Herbst et al., 2014). To date, three immune checkpoint inhibitors (Ipilimumab, Nivolumab, and Pembrolizumab) have been approved for use in SKCM. The main limitation of the use of these immune checkpoint inhibitors is the low response rate (Burtness et al., 2019). We assessed the relationship between FAT10 and immune checkpoints. The results show that the high expression of FAT10 was closely related to PD1, PD-L1, or CTLA-4 in SKCM, suggesting that patients with high expression of FAT10 were more likely to benefit from immunotherapy in SKCM.

In conclusion, our research indicated that FAT10 was highly expressed in multiple types of human cancer (including SKCM) and found to be related to a better prognosis of SKCM. We constructed a reverse mRNA prediction model to reflect the upstream regulatory mechanism of FAT10 in SKCM, namely HCP5/hsa-miR-3127-5p axis. In addition, our current research results showed that FAT10 increased tumor immune cell infiltration and immune checkpoint expression, which was beneficial to tumor immunotherapy, indicating that FAT10 may play its anti-cancer effect in SKCM. However, more basic experiments and clinical trials are needed to confirm our results.

## Data Availability

The datasets presented in this study can be found in online repositories. The names of the repository/repositories and accession number(s) can be found in the article/Supplementary Material.
