# Supplementary figures and images for: FAT10 is a Prognostic Biomarker and Correlated With Immune Infiltrates in Skin Cutaneous Melanoma
Source: Front Mol Biosci. 2022 Mar 1;9:805887. doi: 10.3389/fmolb.2022.805887 (PMC8921645; doi:10.3389/fmolb.2022.805887)

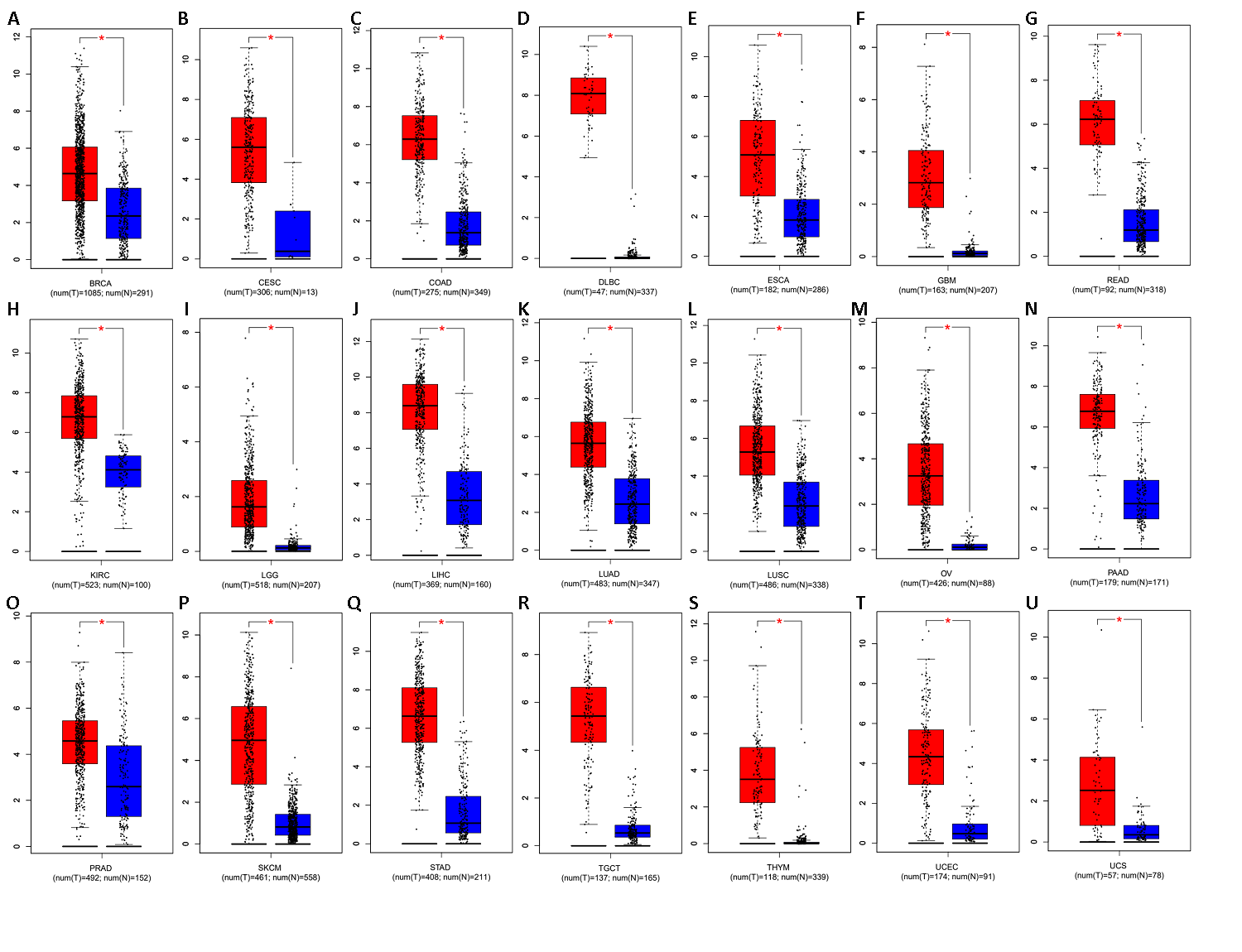

Supplement: Supplementary file 1 [file Image1.TIF]
